# Current status of integrating oncology and palliative care in Japan: a nationwide survey

**DOI:** 10.1186/s12904-020-0515-5

**Published:** 2020-01-24

**Authors:** Y. Uneno, K. Sato, T. Morita, M. Nishimura, S. Ito, M. Mori, C. Shimizu, Y. Horie, M. Hirakawa, T. E. Nakajima, S. Tsuneto, M. Muto

**Affiliations:** 1 Department of Therapeutic Oncology, Graduate School of Medicine, Kyoto University, 54 Kawaharacho Shogoin Sakyo-ku, Kyoto, 606-8507 Japan; 2 Seirei Hospice, Seirei Mikatahara General Hospital, Hamamatsu, Japan; 3 Department of Nursing, Nagoya University Graduate School of Medicine, Nagoya, Japan; 4 Division of Supportive and Palliative Care, Seirei Mikatahara General Hospital, Hamamatsu, Japan; 5 Geriatric Health Service Facility, You-You no Sono, Hiroshima, Japan; 6 Department of Health Informatics, Kyoto University Graduate School of Medicine/ School of Public Health, Kyoto, Japan; 7 Palliative Care Team, Seirei Mikatahara General Hospital, Hamamatsu, Japan; 8 Department of Breast Medical Oncology, National Center for Global Health and Medicine, Tokyo, Japan; 9 Department of Clinical Oncology, St Marianna University School of Medicine, Kawasaki, Japan; 10 Department of Human Health Sciences, Graduate School of Medicine, Kyoto University, Kyoto, Japan

**Keywords:** Palliative care, Oncology, Care delivery, Quality improvement, Organizational innovation

## Abstract

**Background:**

Palliative care (PC) is increasingly recognized as essential for oncology care, and several academic societies strongly recommend integrating oncology and palliative care (IOP) in daily practice. Similarly, the Japanese government encouraged the implementation of IOP through the Cancer Control Act of 2007; however, its detailed progress remains unclear. Therefore, this cross-sectional nationwide survey was conducted to investigate the current status and hospital executive physicians’ perception of IOP.

**Methods:**

The questionnaire was developed based on IOP indicators with international consensus. It was distributed to executive physicians at all government-designated cancer hospitals (DCHs, *n* = 399) and matched non-DCHs (*n* = 478) in November 2017 and the results were compared.

**Results:**

In total, 269 (67.4%) DCHs and 259 (54.2%) non-DCHs responded. The number of PC resources in DCHs was significantly higher than those in non-DCHs (e.g., full-time PC physicians and nurses, 52.8% vs. 14.0%, *p* < 0.001; availability of outpatient PC service ≥3 days per week, 47.6% vs. 20.7%, *p* < 0.001). Routine symptom screening was more frequently performed in DCHs than in non-DCHs (65.1% vs. 34.7%, *p* < 0.001). Automatic trigger for PC referral availability was limited (e.g., referral using time trigger, 14.9% vs. 15.3%, *p* = 0.700). Education and research opportunities were seriously limited in both types of hospitals. Most executive physicians regarded IOP as beneficial for their patients (95.9% vs. 94.7%, *p* = 0.163) and were willing to facilitate an early referral to PC services (54.7% vs. 60.0%, *p* < 0.569); however, the majority faced challenges to increase the number of full-time PC staff, and < 30% were planning to increase the staff members.

**Conclusions:**

This survey highlighted a considerable number of IOP indicators met, particularly in DCHs probably due to the government policy. Further efforts are needed to address the serious research/educational gaps.

## Background

In the last decades, palliative care (PC) is widely recognized as an emerging clinical expertise and an essential part of oncology care [1, 2]. Recent cumulative evidence revealed that early integration of PC is effective for patients with advanced cancer undergoing cancer treatment [3–5]. Several academic societies, including the European Society for Medical Oncology and American Society of Clinical Oncology, strongly support and recommend integrating oncology and palliative care (IOP) in daily oncology practice [6, 7].

Similarly, the Ministry of Health, Labor, and Welfare in Japan has been enhancing the early and continuous delivery of quality care for patients with cancer (both early and advanced stage) from the time point of cancer diagnosis via the Cancer Control Act since 2007 [8, 9]. The policy promotion includes comprehensive strategies including PC training for all physicians working at all government-designated cancer hospitals (DCHs), implementation of PC services and routine screening, the establishment of referral criteria to PC services, and public education [8, 10–13]. However, detailed progress and dissemination of IOP remain unclear.

Therefore, this cross-sectional nationwide survey aimed to investigate the current status and executive physicians’ perception of IOP.

## Methods

This cross-sectional nationwide survey in Japan was conducted targeting executive physicians at hospitals providing cancer treatments as respondents. The survey form was distributed in November 2017, and non-responding institutions were reminded 3 weeks after the first mailing. Responses to the survey in written format were considered consent to participate. Based on the national ethical guideline of epidemiological studies in Japan, this study was exempted from review by the Ethics Committee at the Kyoto University Graduate School and Faculty of Medicine, Kyoto University Hospital.

### Target samples

Two target samplings were identified: one was 399 DCHs, where the Ministry of Health, Labor, and Welfare-authorized high-quality cancer treatment was provided, and the other sample was non-DCHs that potentially manage patients with cancer because a considerable number of patients could receive cancer treatment at non-DCHs. Non-DCHs were randomly sampled and then stratified based on regions and inpatient bed numbers.

**Fig. 1:**
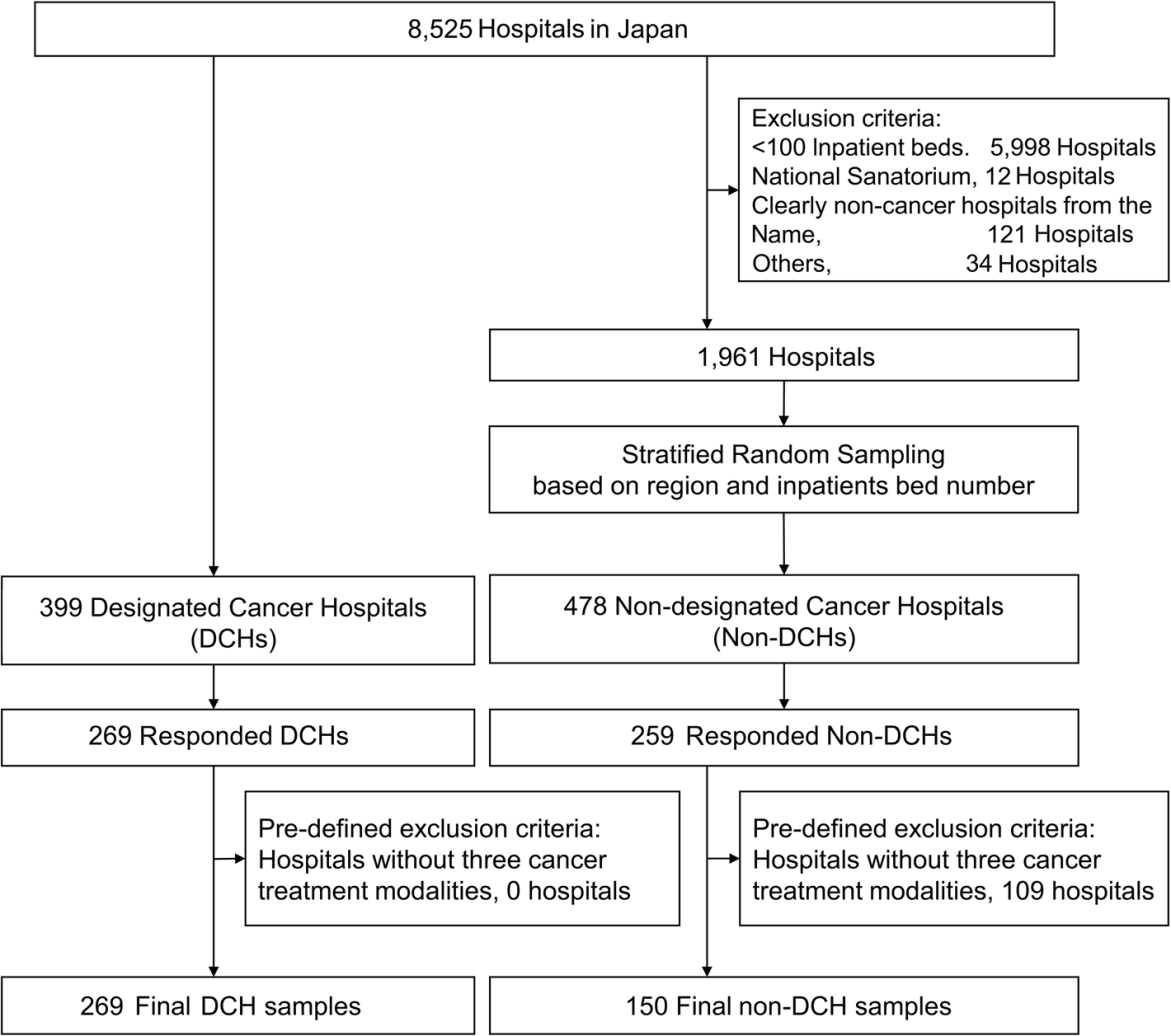
Sampling diagram

The sampling strategy is summarized in Fig. 1. To identify DCHs, the list of DCHs was obtained from the Ministry website as of April 2017. To identify non-DCHs, all hospital data were acquired from Japan Medical Press, Inc., in October 2017. To identify non-DCHs that offer cancer treatment, we excluded the following hospitals: (i) hospitals with < 100 general ward beds (this was because there were no DCHs with < 100 inpatients beds), (ii) national sanatorium, (iii) hospitals not delivering cancer treatment based on the hospital name and clinical departments (e.g., rehabilitation hospitals or no cancer treatment departments), and (iv) others (e.g., breast cancer-specified hospitals). To ensure representativeness and comparability between DCHs and non-DCHs at each region, stratified random sampling was performed based on the region and inpatient bed number. Regional strata were divided into nine categories: Hokkaido, Tohoku, Tokyo, Kanto other than Tokyo, Chubu, Kansai, Chugoku, Shikoku, and Kyushu-Okinawa. Inpatient bed number strata were divided into four categories based on the actual distribution of DCHs: < 299 beds, 300–499 beds, 500–699 beds, and > 700 beds. Considering the possibility that the response rate from non-DCHs may be low, three times more non-DCHs were extracted in each stratum. Moreover, responding hospitals that did not provide three cancer treatment modalities (surgery, systemic chemotherapy, and radiation therapy) at their own hospitals were excluded from the analysis in order to ensure comparability according to predefined exclusion criteria.

### Sample size calculation

We did not define a primary outcome owning to the explanatory nature of the survey. However, to compute the confidence interval of the point estimate within 10%, responses were needed from at least 96 hospitals. Therefore, at least 40% of responses were estimated to be obtained, consisting of 240 hospitals from both types of hospital.

### Survey development

The questionnaire was created after a comprehensive literature search. A pilot test was performed on three physicians with positions equivalent to that of executives in cancer hospitals to ensure face and content validity.

i) Current status of IOP

To clarify the current status of IOP, the international consensus was used as the IOP indicator [14], consisting of five sections: clinical structure, clinical process, clinical outcomes, education, and research, which were categorized as major or minor indicators. Clinical outcome indicators were excluded because the target respondents were experiencing difficulties in answering these questions due to the heterogeneity among specialties within the same hospitals based on the pilot test results. Thus, a total of 25 questions (Tables 2, 3, 4) were primarily used based on the categorical answer format from no (0%), limited (1–24%), approximately half (25–74%), mostly (75–99%), and all (100%) departments.

ii) Executive physicians’ perception toward IOP

To investigate executive physicians’ perceptions of the oncology department toward IOP, 16 questions were used based on the literature search [6, 15–21]. Each question was rated on a 5-point Likert-type scale, from 1 (strongly disagree) to 5 (strongly agree). In addition, a free text query was prepared by asking opinions regarding IOP.

### Analysis

Descriptive statistics was performed to summarize the data. To adjust the biased distribution of inpatient bed number between the responding DCHs and non-DCHs, each stratum of non-DCHs was weighted according to DCH distributions in inpatient bed number (< 299 beds, 300–499 beds, 500–699 beds, and > 700 beds). Missing data was not imputed. The t-test or Cochrane–Armitage trend test was used to compute differences in the proportion, as appropriate. A *P*-value of < 0.05 was considered statistically significant. Owning to the exploratory nature of this survey, the adjustment of multiple testing was not performed. The GNU R software (version 3.2.0; R Project for Statistical Computing, Vienna, Austria) was used for all statistical analyses.

Free comment responses were qualitatively analyzed using inductive content analysis method [22, 23]. Two independent investigators (M.N. and S.I.) reviewed and generated the codes. Then, emerging codes were compared and discussed with an expert PC physician (Y.U.) to achieve agreement of the codes labeled from the data. To ensure rigor and trustworthiness, an experienced investigator (T.M) supervised and examined the consistency of results.

## Results

### Response rates and demographic data

Among the 399 DCHs and 478 non-DCHs surveyed, a total of 269 (67.4%) and 259 (54.2%) responded, respectively. Among the latter, 150 non-DCHs (31.4%) were included for analyses where surgery, chemotherapy, and radiation therapy were performed within the same hospitals to ensure comparability. The hospital’s demographic data are summarized in Table 1.

**Table 1:** Characteristics of responding hospitals

	Unmatched			Matched		
	Designated Cancer Hospitals (*n* = 269)	Non-Designated Cancer Hospitals (*n* = 150)	*P*-value	Designated Cancer Hospitals (*n* = 269)	Non-Designated Cancer Hospitals (*n* = 150)	Adjusted *P*-value
	n (%)	n (%)		n (%)	n (%)	
Inpatient beds number			<.0001			0.998
100–299 beds	16 (5.9)	8 (5.3)		14.2 (5.3)	7.9 (5.3)	
300–499 beds	95 (35.3)	115 (76.7)		96.4 (35.8)	53.7 (35.8)	
500–699 beds	107 (39.8)	23 (15.3)		101.1 (37.6)	56.4 (37.6)	
700 beds	51 (19.0)	4 (2.7)		57.3 (21.3)	32.0 (21.3)	
Activities of responding institutions^a1^						
Inpatient beds available	555.6 [197.8]	414.7 [131.7]	<.0001	563.8 [202.8]	547.5 [234.6]	0.457
Annual number of cancer death	233.3 [158.8]	147.4 [119.1]	<.0001	234.2 [158.5]	201.7 [190.4]	0.078

^a^1: Mean; [], standard deviation

### Major IOP indicators (Table 2)

More than 90% of the DCHs had full-time PC staff (91.5% vs. 42.0%), interdisciplinary PC team (a team of two or more occupations) (98.5% vs. 90.0%), and outpatient clinics (95.2% vs. 58.0%), and the rates were significantly higher than that in non-DCHs. Routine symptom screening was performed in more than half of the DCHs (65.1% vs. 34.7%); however, routine documentation of advanced care planning was performed in < 40% in both types of hospitals (39.0% vs. 28.7%). Automatic trigger for PC referral was also employed in < 40% of surveyed hospitals (time trigger, 14.9% vs. 15.3%; needs trigger, 37.5% vs. 27.3%).

**Table 2:** Current status of major indicators in the integration of oncology and palliative care programs in Japan

	Designated Cancer Hospitals (*n* = 269)	Non-Designated Cancer Hospitals (*n* = 150)	*P* value	Adjusted *P* value
	n (%)	n (%)		
Clinical structure				
Presence of palliative care inpatient consultation team			< 0.001	< 0.001
involving full-time both physicians and nurses	142 (52.8)	21 (14.0)		
involving full-time physicians only	1 (0.4)	5 (3.3)		
involving full-time nurses only	103 (38.3)	37 (24.7)		
involving part-time both physicians and nurses	17 (6.3)	68 (45.3)		
not available	1 (0.4)	17 (11.3)		
Presence of palliative care outpatient clinic			< 0.001	< 0.001
available ≥5 days a week	85 (31.6)	16 (10.7)		
available 3–4 days a week	43 (16.0)	15 (10.0)		
available < 1–2 days a week	128 (47.6)	56 (37.3)		
not available	7 (2.6)	60 (40.0)		
Clinical process				
Presence of interdisciplinary palliative care team^a1^	265 (98.5)	135 (90.0)	< 0.001	0.015
Members of palliative care team				
Pain clinicians or anesthesiologists	117 (43.5)	40 (26.7)	< 0.001	0.024
Palliative care physicians expecting pain clinicians or anesthesiologists	225 (83.6)	122 (81.3)	0.61	0.208
Palliative care nurses	265 (98.5)	132 (88.0)	< 0.001	< 0.001
Healthcare professionals treating psychological issues (e.g., psychotherapist, psychiatrist, chaplain, or social worker)	247 (91.8)	100 (66.7)	< 0.001	< 0.001
Medical social workers	215 (79.9)	93 (62.0)	< 0.001	0.008
Nutritionists	181 (67.2)	88 (58.7)	0.085	0.435
Pharmacists	259 (96.3)	133 (88.7)	0.001	0.024
Others	100 (37.2)	40 (26.7)	0.031	0.028
Routine symptom screening in the outpatient oncology clinic			< 0.001	0.003
All departments (100%)	78 (29.0)	21 (14.0)		
Most departments (75–99%)	62 (23.0)	21 (14.0)		
Approximately half departments (25–74%)	35 (13.0)	10 (6.7)		
Limited departments (1–24%)	71 (26.4)	41 (27.3)		
No department (0%)	19 (7.1)	53 (35.3)		
Routine documentation of advance care plans in patients with advanced cancer			0.050	0.183
All departments (100%)	40 (14.9)	17 (11.3)		
Most departments (75–99%)	42 (15.6)	14 (9.3)		
Approximately half departments (25–74%)	23 (8.6)	12 (8.0)		
Limited departments (1–24%)	47 (17.5)	31 (20.7)		
No department (0%)	111 (41.3)	71 (47.3)		
Early referral to palliative care using time trigger (e.g., 3 months after the diagnosis of incurability)			0.700	0.358
All departments (100%)	12 (4.5)	7 (4.7)		
Most departments (75–99%)	13 (4.8)	5 (3.3)		
Approximately half departments (25–74%)	15 (5.6)	8 (5.3)		
Limited departments (1–24%)	26 (9.7)	14 (9.3)		
No department (0%)	199 (74.0)	113 (75.3)		
Early referral to palliative care using needs trigger (e.g., pain with NRS ≥7)			0.030	0.820
All departments (100%)	49 (18.2)	19 (12.7)		
Most departments (75–99%)	35 (13.0)	10 (6.7)		
Approximately half departments (25–74%)	18 (6.7)	12 (8.0)		
Limited departments (1–24%)	20 (7.4)	16 (10.7)		
No department (0%)	143 (53.2)	90 (60.0)		

^a^1, a team of two or more occupations

### Minor IOP indicators (Table 3)

Symptom management guidelines (88.9% vs. 78.7%) and PC referral criteria (71.7% vs. 58.7%) were well equipped in both types of hospitals. Concurrent services between oncology and PC were available in more than 95% of hospitals (97.8% vs. 96.7%), and more than half of them (86.3% vs. 53.3%) involved the PC staff in the multidisciplinary tumor conference. With regard to prompt PC service delivery, both types of hospitals tend to have less capacity in outpatient setting compared to inpatient settings (inpatient settings, 93.7% vs. 73.3%; outpatient settings, 78.8% vs. 60.7%).

**Table 3:** Current status of minor indicators in the integration of oncology and palliative care programs in Japan

	Designated Cancer Hospitals (*n* = 269)	Non-Designated Cancer Hospitals (*n* = 150)	*P*-value	Adjusted *P*-value
	n (%)	n (%)		
Clinical Process				
Institutionally accepted palliative care symptom management guidelines in written format	239 (88.9)	118 (78.7)	0.004	0.238
Institutionally accepted palliative care referral criteria available in written format	193 (71.7)	88 (58.7)	< 0.001	0.077
Available institutionally accepted clinical care pathways (automatic triggers) for palliative care referral	47 (17.8)	8 (5.3)	< 0.001	< 0.001
Palliative care team routinely involved in multidisciplinary tumor conference for patient case discussions				
Attending always	128 (47.6)	38 (25.3)	< 0.001	< 0.001
Attending when necessary	104 (38.7)	42 (28.0)		
Not attending	32 (11.9)	22 (14.7)		
Multidisciplinary tumor conference is not held/no palliative care team	3 (1.1)	46 (30.7)		
Presence of palliative care specialists among cancer center senior leadership (e.g., head of oncology department/division and chief executives)	103 (38.3)	44 (29.3)	< 0.001	0.052
Administration of systemic cancer therapy (e.g., chemotherapy and targeted agents) in palliative care patients possible	263 (97.8)	145 (96.7)	0.115	0.262
Availability of the same-day inpatient palliative care consultation upon request				
Almost all (≥90%)	192 (71.4)	69 (46.0)	< 0.001	< 0.001
Mostly (50–89%)	60 (22.3)	41 (27.3)		
Less likely (< 49%)	14 (5.2)	28 (18.7)		
Not available	0 (0.0)	12 (8.0)		
Availability of same-day outpatient palliative care consultation upon request				
Almost all (≥90%)	140 (52.0)	42 (28.0)	< 0.001	< 0.001
Mostly (50–89%)	72 (26.8)	49 (32.7)		
Less likely (< 49%)	52 (19.3)	35 (23.3)		
Not available	2 (0.7)	23 (15.3)		

### Education and research IOP indicators (Table 4)

Education opportunity of PC for oncologists was limited in both types of hospitals (e.g., routine rotation in PC, 8.2% vs. 2.0%); and education opportunity on oncology for PC physicians was also limited (e.g., routine rotation in oncology, 9.7% vs. 1.3%). Research opportunity was more frequent in DCHs, but still far limited in both types of hospitals (e.g., tenured faculty in PC, 3.3% vs. 1.3%).

**Table 4:** Current status of education and research indicators in the integration of oncology and palliative care programs in Japan

	Designated Cancer Hospitals (*n* = 269)	Non-Designated Cancer Hospitals (*n* = 150)	*P*-value	Adjusted *P*-value
	n (%)	n (%)		
Education, major indicators				
Didactic palliative care curriculum for oncology fellows			< 0.001	0.195
≥ 50% fellows attend	63 (23.4)	14 (9.3)		
< 50% fellows attend	78 (29.0)	39 (26.0)		
Not available for the palliative care education	124 (46.1)	95 (63.3)		
Oncology fellows have routine rotation in palliative care			0.007	0.224
≥ 50% fellows have	22 (8.2)	3 (2.0)		
< 50% fellows have	26 (9.7)	12 (8.0)		
Not available for the palliative care education	217 (80.7)	133 (88.7)		
Combined palliative care and oncology educational activities for fellows/trainees			0.002	0.394
≥ 50% fellows/trainees attend	20 (7.4)	6 (4.0)		
< 50% fellows/trainees attend	69 (25.7)	25 (16.7)		
Not available for the palliative care education	174 (64.7)	117 (78.0)		
Continuing medical education in palliative care for attending oncologists			0.002	0.260
≥ 50% attending oncologists attend	28 (10.4)	7 (4.7)		
< 50% attending oncologists attend	108 (40.1)	47 (31.3)		
Not available for the palliative care education	129 (48.0)	94 (62.7)		
Education, minor indicators				
Palliative care fellows have routine rotation in oncology			< 0.001	0.011
≥ 50% fellows attend	26 (9.7)	2 (1.3)		
< 50% fellows attend	20 (7.4)	10 (6.7)		
Not available for the palliative care education	220 (81.8)	137 (91.3)		
Continuing medical education in oncology for palliative care specialists			0.003	0.376
≥ 50% specialists attend	36 (13.4)	12 (8.0)		
< 50% specialists attend	34 (12.6)	11 (7.3)		
Not available for the palliative care education	197 (73.2)	126 (84.0)		
Research				
Institutional funding for palliative oncology research	50 (18.6)	11 (7.3)	0.002	0.039
Peer-reviewed publications in palliative oncology	58 (21.6)	18 (12.0)	0.015	0.052
Tenured faculty in palliative care	9 (3.3)	2 (1.3)	0.213	0.312
Collaborative research between oncology and palliative care	29 (10.8)	13 (8.7)	0.467	0.858

### Executive physicians’ perspectives toward IOP (Additional file 1)

A total of ≥70% executive physicians in both types of hospitals did not regard their primary PC (PC services which was provided by the primary physicians and nurses) as good enough (74.7% vs. 75.3%). They also indicated that IOP encouragement would be beneficial for their patients (95.9% vs. 94.7%) and not be costly for their hospitals (29.4% vs. 33.3%). However, many hospitals were facing challenges to allocate more staff to provide PC services (75.5% vs. 86.7%). More than half of hospitals were planning to facilitate early referral to PC services (54.6% vs. 60.0%).

### Qualitative analysis of free text query

In total, 106 (26.6%) DCHs and 68 (14.2%) non-DCHs responded to the free text query. Summarized data is presented in Additional files 2, 3 and 4. Three major categories were generated: perception toward IOP, challenges to encourage IOP, and solutions to encourage IOP. The typical perception was that enhancing the primary PC service is vital due to the large estimated number of patients with PC needs as compared with the available specialized PC service, although the importance of IOP, in general, was recognized.

## Discussion

This nationwide survey comprehensively investigated the current status and executive physicians’ perspectives of IOP in Japan. From 2007, the Ministry of Health, Labor, and Welfare has presented several mandatory requirements for DCH certification, such as referral criteria for PC services, institutionally accepted symptom management guidelines, and routine PC screening, and many of these requirements overlapped with the surveyed IOP indicators. Thus, the current IOP status at DCHs was found to be highly satisfied with respect to the clinical structure and process indicators even as compared with the previous literature in the European and North American countries [6, 15, 17–20]. For instance, outpatient PC service availability was equivalent to European Society for Medical Oncology (ESMO)-designated centers (DCs) (e.g., Japan DCHs vs. ESMO DCs: outpatient clinic available 95% vs. 89%; same-day outpatient consultation available, 79% vs. 82%) [6]. This supported the promising role of the government to disseminate quality care nationwide.

Our survey also found that research and education opportunities were seriously limited at the vast majority of cancer hospitals. Continuing education for attending physicians was held in more than half of the facilities in ESMO DCs, whereas more than half of Japanese hospitals did not have opportunities for continuing education [6]. Mutual rotation training opportunities for fellows were also limited between oncology and PC departments. Research infrastructure was also extremely limited, with levels similar to those in the USA in 2010 [15] (e.g., institutional funding for palliative oncology research, 19% in Japan vs. 13% in the USA). As education and research activities across the specialty can promote smooth coordination among healthcare professionals [24–26], further efforts to develop education and research infrastructures (e.g. employing tenured or full-time PC faculty who engages the education and research) are valuable.

Most leaders in cancer hospitals recognized IOP as beneficial and reasonable for their cancer patients. They also planned to facilitate early referral of cancer patients to PC services. Meanwhile, they were facing challenges in recruiting specialized PC staff and less likely planned to employ more PC staff. Accordingly, the current specialized PC staff seemed to be overwhelmed by the routine clinical practice and had actual difficulties to implement more IOPs. Lack of PC resources is a well-known barrier for IOP, and our qualitative analysis also supported this situation [27–29]. Given that enrichment of PC resources is unrealistic, enhancing the primary PC provided by oncologists to effectively optimize existing resources such as standardized care pathway may be valuable [1].

This study had several limitations. First, as target respondents were executive physicians in oncology departments, it was not evident whether several IOP indicators actually work in routine practice. Detailed analysis of how those indicators work in practice would help determine the optimal clinical IOP models. Second, as the target hospitals were restricted to those providing three cancer treatment modalities (i.e., surgery, radiation therapy, and chemotherapy), the results in these analyses cannot be generalized in smaller hospitals. Third, owning to the exploratory nature of the study, multiple tests were not adjusted which could limit the implications of the detected significant differences.

## Conclusions

This survey highlighted that a considerable number of IOP indicators were satisfied in DCHs in Japan. Further investigation is warranted to clarify whether these indicators effectively work in achieving real clinical situations.

## Supplementary information

**Additional file 1: TableS1.** Perception of the integration of oncology and palliative care programs in Japan

**Additional file 2: TableS2.** Qualitative analysis of opinions toward the integration of oncology and palliative care

**Additional file 3: TableS3.** Challenges to encourage IOP

**Additional file 4: TableS4.** Solutions to encourage IOP

## Data Availability

The datasets used and/or analysed during the current study are available from the corresponding author on reasonable request.

## Abbreviations

DC: Designated center
DCH: Government-designated cancer hospital
ESMO: European Society for Medical Oncology
IOP: Integrating oncology and palliative care
PC: Palliative care
