# Supplementary material for: Current status of integrating oncology and palliative care in Japan: a nationwide survey
Source: BMC Palliat Care. 2020 Jan 24;19:12. doi: 10.1186/s12904-020-0515-5 (PMC6982384; doi:10.1186/s12904-020-0515-5)
Supplement: Supplementary file 1 — Additional file 1: TableS1. Perception of the integration of oncology and palliative care programs in Japan [file 12904_2020_515_MOESM1_ESM.docx]

|  | Designated Cancer Hospitals (n = 269) | Non-Designated Cancer Hospitals  (n = 150) | *P*-value | Adjusted *P*-value |
| --- | --- | --- | --- | --- |
|  | n (%) | n (%) |  |  |
| Primary palliative care services provided by my institution provide are good enough |  |  |  |  |
| - Strongly agree | 1 (0.3) | 2 (1.3) | 0.716 | 0.674 |
| - Agree | 22 (8.2) | 13 (8.7) |  |  |
| - Partially agree | 41 (15.2) | 21 (14.0) |  |  |
| - Disagree | 150 (55.8) | 76 (50.7) |  |  |
| - Strongly disagree | 51 (19.0) | 37 (24.7) |  |  |
| Facilitating integration of oncology and palliative care programs will benefit patients at my institution | |  |  |  |
| - Strongly agree | 131 (48.7) | 81 (54.0) | 0.163 | 0.933 |
| - Agree | 96 (35.7) | 52 (34.7) |  |  |
| - Partially agree | 31 (11.5) | 9 (6.0) |  |  |
| - Disagree | 6 (2.2) | 2 (1.3) |  |  |
| - Strongly disagree | 2 (0.7) | 2 (1.3) |  |  |
| Facilitating integration of oncology and palliative care programs will be costly for my institution | |  |  |  |
| - Strongly agree | 7 (2.6) | 8 (5.3) | 0.764 | 0.217 |
| - Agree | 26 (9.7) | 19 (12.7) |  |  |
| - Partially agree | 46 (17.1) | 23 (15.3) |  |  |
| - Disagree | 146 (54.3) | 61 (40.7) |  |  |
| - Strongly disagree | 41 (15.2) | 37 (24.6) |  |  |
| Palliative care team at my institutions cannot provide more effort to enhance integration of oncology and palliative care programs besides the current routine practice | | | | |
| - Strongly agree | 29 (10.8) | 24 (16.0) | 0.103 | 0.292 |
| - Agree | 65 (24.2) | 35 (23.3) |  |  |
| - Partially agree | 84 (31.2) | 44 (29.3) |  |  |
| - Disagree | 74 (27.5) | 38 (25.3) |  |  |
| - Strongly disagree | 15 (5.6) | 4 (2.7) |  |  |
| Allocating more staff to the palliative care services is challenging at my institution |  |  |  |  |
| - Strongly agree | 35 (13.0) | 32 (21.3) | 0.001 | 0.002 |
| - Agree | 83 (30.9) | 56 (37.3) |  |  |
| - Partially agree | 85 (32) | 42 (28.0) |  |  |
| - Disagree | 44 (16.4) | 12 (8.0) |  |  |
| - Strongly disagree | 20 (7.4) | 7 (4.7) |  |  |
| Currently, increasing physicians’ full-time palliative care is challenging at my institution | |  |  |  |
| - Strongly agree | 75 (27.9) | 82 (54.7) | <0.001 | <0.001 |
| - Agree | 58 (21.6) | 38 (25.3) |  |  |
| - Partially agree | 39 (14.5) | 10 (6.7) |  |  |
| - Disagree | 30 (11.1) | 8 (5.3) |  |  |
| - Strongly disagree | 65 (24.2) | 9 (6.0) |  |  |
| My institution will be increasing the number of full-time palliative care physicians over the next 5 years | 103 (38.3) | 42 (28.0) | 0.023 | 0.852 |
| Currently, increasing full-time palliative care nurses is challenging at my institution |  |  |  |  |
| - Strongly agree | 8 (3.0) | 21 (14.0) | <0.001 | <0.001 |
| - Agree | 28 (10.4) | 42 (28.0) |  |  |
| - Partially agree | 38 (14.1) | 27 (18.0) |  |  |
| - Disagree | 60 (22.3) | 27 (18.0) |  |  |
| - Strongly disagree | 133 (49.4) | 32 (21.3) |  |  |
| My institution will be increasing the number of full-time palliative care nurses over the next 5 years | 93 (34.6) | 47 (31.3) | 0.423 | 0.477 |
| Currently, increasing full-time staff who care for patients with psychological issues is challenging at my institution (e.g., Psychotherapist, Psychiatrist, Religionist) | | | | |
| - Strongly agree | 30 (11.1) | 34 (22.7) | <0.001 | 0.077 |
| - Agree | 44 (16.4) | 28 (18.7) |  |  |
| - Partially agree | 47 (17.8) | 30 (20.0) |  |  |
| - Disagree | 73 (27.1) | 35 (23.3) |  |  |
| - Strongly disagree | 70 (26.0) | 21 (14.0) |  |  |
| My institution will be increasing full-time staff who care for patients with the psychological issues over the next 5 years | 62 (23.0) | 30 (20.0) | 0.247 | 0.598 |
| My institution will be increasing/developing palliative care beds over the next 5 years | 41 (15.2) | 19 (12.7) | 0.442 | 0.929 |
| My institution will be increasing funding for palliative care over the next 5 years | 50 (18.6) | 25 (16.7) | 0.569 | 0.542 |
| My institution will be facilitating early referral to palliative care over the next 5 years | 147 (54.6) | 90 (60.0) | 0.352 | <0.001 |
| My institution will use time trigger for early referral to palliative care  (e.g., 3 months after diagnosis of incurability) | 82 (30.5) | 58 (38.7) | 0.099 | 0.489 |
| My institution will use needs trigger for early referral to palliative care  (e.g., pain with NRS of ≥7) | 137 (50.9) | 81 (54.0) | 0.554 | 0.063 |
