# Supplementary material for: Current status of integrating oncology and palliative care in Japan: a nationwide survey
Source: BMC Palliat Care. 2020 Jan 24;19:12. doi: 10.1186/s12904-020-0515-5 (PMC6982384; doi:10.1186/s12904-020-0515-5)
Supplement: Supplementary file 2 — Additional file 2: TableS2. Qualitative analysis of opinions toward the integration of oncology and palliative care [file 12904_2020_515_MOESM2_ESM.docx]

|  |  | *n* |
| --- | --- | --- |
| **Perception toward IOP** | |  |
| **Negative** |  |  |
|  | Providing PC is the fundamental role of medical oncologists. | 22 |
|  | Not all patients need early specialized PC. | 21 |
|  | Considerable barriers exist to implement EPC. | 21 |
|  | Specialized PC staff not needed to provide primary level PC | 14 |
|  | EPC increases too much burden for the PC department. | 10 |
|  | No consensus toward uniform definition of EPC | 7 |
| **Positive** |  |  |
|  | EPC is a clinically warranted issue. | 32 |
|  | Considerable need for EPC exists | 5 |
| **Challenges to encourage IOP** | |  |
| **Clinical Resources** |  |  |
|  | Lack of PC staff | 71 |
|  | Concurrent post for PC staff | 19 |
|  | Instable financial status of the hospital | 17 |
|  | Epidemiological maldistribution of clinical resources | 10 |
|  | Insufficient educational recourses | 9 |
| **Clinical Process** |  |  |
|  | Lack of standardized referral criteria | 7 |
|  | No definite practical model of EPC | 6 |
|  | Heterogeneity of practice patterns among HCPs | 5 |
| **Patient & Family** |  |  |
|  | Reluctance to receive PC | 25 |
|  | Lack of optimal understanding toward PC | 9 |
| **Oncology Staff** |  |  |
|  | Lack of optimal understanding toward PC | 35 |
| **Hospital heads** |  |  |
|  | Lack of optimal understanding toward PC | 17 |
| **Policy Makers** |  |  |
|  | Inappropriate reimbursement for PC | 13 |
| **Solutions to encourage IOP** | |  |
| **Clinical Resources** | |  |
|  | Increasing PC staff | 21 |
|  | Securing full-time post for PC department | 11 |
| **Clinical Process** | |  |
|  | Multidisciplinary care | 25 |
|  | Screening of patient needs and symptoms | 20 |
|  | Optimal way to identify patients with PC needs | 17 |
|  | Active communication among HCPs | 10 |
|  | Coordination with other community recourses | 5 |
| **Policy Maker** |  |  |
|  | Optimization of reimbursement for PC | 11 |
| **Patient/citizen education** | |  |
|  | Changing the image of PC | 16 |
|  | Enlightenment of optimal understanding of PC | 15 |
|  | Emphasize the benefits of EPC | 14 |
| **HCPs perceptions** | |  |
|  | Understanding the importance of PC | 16 |
|  | Understanding that PC is a natural part of oncology care | 8 |
|  | Attitude to identify patients with PC needs | 7 |
| **Hospital heads** | |  |
|  | Understanding the importance of PC | 7 |
| **HCPs education** | |  |
|  | Primary PC education | 38 |
|  | Specialized PC education | 24 |
|  | Continuous PC education | 11 |
|  | Undergraduate PC education | 8 |
|  | Residents/fellows PC education | 7 |
|  | Sharing experience of successful PC practice | 5 |
| abbreviation: PC, palliative care; HCP healthcare professional; EPC, early palliative care. Opinions that more than five hospitals replied are displayed. | | |
