# Supplementary material for: Current status of integrating oncology and palliative care in Japan: a nationwide survey
Source: BMC Palliat Care. 2020 Jan 24;19:12. doi: 10.1186/s12904-020-0515-5 (PMC6982384; doi:10.1186/s12904-020-0515-5)
Supplement: Supplementary file 3 — Additional file 3: TableS3. Challenges to encourage IOP [file 12904_2020_515_MOESM3_ESM.docx]

|  | | *n* |
| --- | --- | --- |
| **Clinical Resources** |  |  |
|  | Lack of PC staff | 71 |
|  | Concurrent post for PC staff | 19 |
|  | Unstable financial status of the hospital | 17 |
|  | Epidemiological maldistribution of clinical resources | 10 |
|  | Insufficient educational recourses | 9 |
| **Clinical Process** |  |  |
|  | Lack of standardized referral criteria | 7 |
|  | No definite practical model of EPC | 6 |
|  | Heterogeneity of practice patterns among HCPs | 5 |
| **Patient & Family** |  |  |
|  | Reluctance to receive PC | 25 |
|  | Lack of optimal understanding toward PC | 9 |
| **Oncology Staff** |  |  |
|  | Lack of optimal understanding toward PC | 35 |
| **Hospital heads** |  |  |
|  | Lack of optimal understanding toward PC | 17 |
| **Policy Makers** |  |  |
|  | Inappropriate reimbursement for PC | 13 |
| abbreviation: PC, palliative care; EPC, early palliative care; HCP, healthcare professional | | |
