# Supplementary material for: Current status of integrating oncology and palliative care in Japan: a nationwide survey
Source: BMC Palliat Care. 2020 Jan 24;19:12. doi: 10.1186/s12904-020-0515-5 (PMC6982384; doi:10.1186/s12904-020-0515-5)
Supplement: Supplementary file 4 — Additional file 4: TableS4. Solutions to encourage IOP [file 12904_2020_515_MOESM4_ESM.docx]

|  | | *n* |
| --- | --- | --- |
| **Clinical Resources** |  |  |
|  | Increasing PC staff | 21 |
|  | Securing full-time post for the PC department | 11 |
| **Clinical Process** |  |  |
|  | Multidisciplinary care | 25 |
|  | Screening of patient needs and symptoms | 20 |
|  | Optimal way to identify patients with PC needs | 17 |
|  | Active communication among HCPs | 10 |
|  | Coordination with other community recourses | 5 |
| **Policy Maker** |  |  |
|  | Optimization of reimbursement for PC | 11 |
| **Pt/citizen education** |  |  |
|  | Changing the image of PC | 16 |
|  | Enlightenment of optimal understanding of PC | 15 |
|  | Emphasizing the benefits of EPC | 14 |
| **HCPs perceptions** |  |  |
|  | Understanding the importance of PC | 16 |
|  | Understanding that PC as a natural part of oncology care | 8 |
|  | Attitude to identify patients with PC needs | 7 |
| **Hospital heads** |  |  |
|  | Understanding the importance of PC | 7 |
| **HCPs education** |  |  |
|  | Primary PC education | 38 |
|  | Specialized PC education | 24 |
|  | Continuous PC education | 11 |
|  | Undergraduate PC education | 8 |
|  | Residents/fellows PC education | 7 |
|  | Sharing experience of successful PC practice | 5 |
| abbreviation: PC, palliative care; HCP, healthcare professional; EPC, early palliative care | | |
